# Efficient framework for predicting MiRNA-disease associations based on improved hybrid collaborative filtering

**DOI:** 10.1186/s12911-021-01616-5

**Published:** 2021-08-30

**Authors:** Ru Nie, Zhengwei Li, Zhu-hong You, Wenzheng Bao, Jiashu Li

**Affiliations:** 1 Engineering Research Center of Mine Digitalization of Ministry of Education, China University of Mining and Technology, Xuzhou, 221116 China; 2 School of Computer Science and Technology, China University of Mining and Technology, Xuzhou, 221116 China; 3 Institute of Machine Learning and Systems Biology, College of Electronics and Information Engineering, Tongji University, Shanghai, 201804 China; 4 KUNPAND Communications (Kunshan) Co., Ltd., Suzhou, 215300 China; 5 School of Computer Science, Northwestern Polytechnical University, Xi’an, 710072 China; 6 School of Information Engineering, Xuzhou University of Technology, Xuzhou, 221018 China

**Keywords:** miRNA-disease association prediction, Hybrid collaborative filtering, Heterogeneous data, Singular value decomposition

## Abstract

**Background:**

Accumulating studies indicates that microRNAs (miRNAs) play vital roles in the process of development and progression of many human complex diseases. However, traditional biochemical experimental methods for identifying disease-related miRNAs cost large amount of time, manpower, material and financial resources.

**Methods:**

In this study, we developed a framework named hybrid collaborative filtering for miRNA-disease association prediction (HCFMDA) by integrating heterogeneous data, e.g., miRNA functional similarity, disease semantic similarity, known miRNA-disease association networks, and Gaussian kernel similarity of miRNAs and diseases. To capture the intrinsic interaction patterns embedded in the sparse association matrix, we prioritized the predictive score by fusing three types of information: similar disease associations, similar miRNA associations, and similar disease-miRNA associations. Meanwhile, singular value decomposition was adopted to reduce the impact of noise and accelerate predictive speed.

**Results:**

We then validated HCFMDA with leave-one-out cross-validation (LOOCV) and two types of case studies. In the LOOCV, we achieved 0.8379 of AUC (area under the curve). To evaluate the performance of HCFMDA on real diseases, we further implemented the first type of case validation over three important human diseases: Colon Neoplasms, Esophageal Neoplasms and Prostate Neoplasms. As a result, 44, 46 and 44 out of the top 50 predicted disease-related miRNAs were confirmed by experimental evidence. Moreover, the second type of case validation on Breast Neoplasms indicates that HCFMDA could also be applied to predict potential miRNAs towards those diseases without any known associated miRNA.

**Conclusions:**

The satisfactory prediction performance demonstrates that our model could serve as a reliable tool to guide the following research for identifying candidate miRNAs associated with human diseases.

## Introduction

MicroRNAs (miRNAs) are endogenous small noncoding RNAs (19–22 nucleotides) that could regulate gene expression by base-pairing to partially complementary mRNAs [1]. Since the first miRNA, lin-4, was discovered by Lee et al*.* in 1993 [2], more than 38,000 miRNA sequences from 271 organisms have been accumulated to date [3]. Plenty of evidence indicates that miRNAs play critical roles in many fundamental and important biological processes, such as immune response, transcription, proliferation and differentiation [4]. The mutation and dysregulated expression of miRNAs may be connected with the development and progression of many diseases [5, 6]. For instance, miR-155 downregulated target gene TP53INP1 whose expression was strongly reduced in pancreatic ductal adenocarcinoma development [7]. Besides, induction of endogenous miR‐340 expression was capable to suppress tumor cell migration and invasion, whereas miR‐340 knockdown led to breast cancer cell migration and invasion [8]. Moreover, three most up-regulated miRNAs (miR-221, 222, and 146) distinguished unequivocally between papillary thyroid carcinoma and normal thyroid [9]. Therefore, exploring the relationships between miRNAs and diseases could not only provide novel insights into disease pathogenesis at the molecular level, but also benefit the design of specific molecular tools for disease diagnosis, treatment and prevention [10, 11].

However, traditional in vivo or biochemical experiment for identifying disease-related miRNA candidates have multiple bottlenecks, such as long operation time, extremely high cost and false positive results [12, 13]. Consequently, quickly and automatically identifying these associations with in silico methods is a useful supplement for future experimental validation and could substantially reduce the cost and effort [14–16]. Actually, based on the generally accepted assumption that functionally similar miRNAs are likely to be associated with phenotypically similar diseases and vice versa, a large number of computational models have been proposed for identifying potential disease-related miRNAs in recent years. For example, Jiang et al*.* [17] explored a network-based computational model through hypergeometric distribution to prioritize disease-related miRNAs. Shi et al*.* [18] focused on the functional connections between miRNA targets and disease genes in protein–protein interaction (PPI) networks and presented a novel method to identify disease-related miRNAs. Xu et al*.* [19] introduced an approach based on MTDN for prioritizing putative miRNAs associated with diseases by combining paired miRNA and mRNA expression data. In addition, Chen et al*.* [20] constructed HGIMDA framework to identify potential disease-related miRNAs by combing multiple source information, e.g., experimentally validated miRNA-disease relationship, disease semantic similarity, miRNA functional similarity, Gaussian interaction profile kernel similarity. Based on the same multiple source data, Chen et al*.* [21] also presented PRMDA to infer potential disease-related miRNAs by personalized recommendation-based algorithm. Although HGIMDA and PRMDA could be applied to those diseases without experimentally validated miRNA, the predictive accuracy needs to be further enhanced. In addition, Marissa et al*.* [22] presented an in-silico method named MAP for predicting putative miRNA-disease associations through network diffusion on multi-omics biological data including miRNA-gene associations, protein–protein interactions, and gene-disease associations, and so on. Yu et al*.* [23] proposed TCRWMDA for miRNA-disease association prediction through three-layer heterogeneous network combined with unbalanced random walk. The case study results indicate that TCRWMDA is an effective tool to predict the potential miRNA-disease associations. Li et al. [24]*.* developed a novel method named NIMCGCN which employs graph convolutional networks to extract feature representations and a neural inductive matrix completion model to generate association matrix completion. Experimental results indicate this method could be used for predicting those diseases without any known related miRNAs.

Collaborative filtering aims at predicting the user interest for a given item based on a collection of user profiles and there are already some basic applications in miRNA-disease associations (MDAs) prediction [25–27]. However, these approaches generally fail to achieve satisfactory results. In this work, we developed a computational approach named hybrid collaborative filtering for predicting miRNA-disease associations (HCFMDA) to infer putative associations between diseases and miRNAs. By fusing experimentally verified MDAs, disease similarity, and miRNA functional similarity to mine intrinsic discriminative information embedded in the correlations between diseases and miRNAs, HCFMDA could be applied for identifying potential miRNAs for those diseases without any known related miRNA. In the leave-one-out cross-validation (LOOCV), HCFMDA achieved AUC (area under the curve) value of 0.8379 and demonstrated reliable predictive performance. In addition, we also used HCFMDA to carry out two types of case validation on four important human complex diseases (Colon Neoplasms, Esophageal Neoplasms, Prostate Neoplasms and Breast Neoplasms). As a result, 88%, 92%, 88% and 92% out of the top 50 putative miRNAs for those 4 diseases were confirmed by experiment evidence. All the results indicate HCFMDA is effective and reliable for the prediction of MDAs.

## Materials and methods

### Human miRNA-disease associations

The experimentally verified human miRNA-disease associations for HCFMDA were retrieved from HMDD v2.0 database [28]. After the data preprocessing and verification, we obtained altogether 5430 experimentally validated associations between 383 diseases and 495 miRNAs. Then, we constructed an adjacency matrix $X \in {\text{R}}^{{N_{d} \times N_{m} }}$ to represent the corresponding associations, where $N_{d}$ and $N_{m}$ are the number of the diseases and miRNAs, respectively. Here, element ${\text{X}}_{ij}$ in the matrix is 1 or 0, with 1 representing a known association between disease $d_{i}$ and miRNA $m_{j}$, and 0 denoting an unknown one. Correspondingly, the matrix $X$ could be decomposed into row vectors:

1 $$X = \left[ {r_{1} , \ldots ,r_{{N_{d} }} } \right]^{T} , r_{i} = \left[ {r_{i,1} , \ldots ,r_{{i,N_{m} }} } \right],\quad i = 1, \ldots ,N_{d}$$

where $T$ denotes transpose operation and row vector $r_{i}$ represents the interaction profile of disease $d_{i}$. As described below, this representation was mainly used for disease-based collaborative filtering. Alternatively, the matrix $X$ could also be decomposed into column vectors:

2 $$X = \left[ {c_{1} , \ldots ,c_{{N_{m} }} } \right], c_{j} = \left[ {c_{j,1} , \ldots ,c_{{j,N_{d} }} } \right]^{T} , \quad j = 1, \ldots ,N_{m}$$

where column vector $c_{j}$ corresponds to miRNA $m_{j}$. Likewise, this representation could be used for miRNA-based collaborative filtering.

### MiRNA functional similarity

Based on the hypothesis that miRNAs with similar functions tend to be related to similar disease phenotypes, Wang et al*.* pioneered the human miRNA functional similarity which is available at http://www.cuilab.cn/files/images/cuilab/misim.zip [29]. We herein constructed matrix $FM$ to express their functional similarity scores, where the entity $FM_{ij}$ denotes the similarity of miRNA pair $\left\langle {m_{i} ,m_{j} } \right\rangle$.

### Disease semantic similarity

According to previous study [30], we introduced directed acyclic graph (DAG) to express disease based on the Medical Subject Headings (MeSH) descriptors of category C from http://www.nlm.nih.gov/. Disease $d_{i}$ could be denoted as $DAG\left( {d_{i} } \right) = \left( {V\left( {d_{i} } \right),E\left( {d_{i} } \right)} \right)$, where $V\left( {d_{i} } \right)$ is a set consisting all ancestral nodes of $d_{i}$ and $d_{i}$ itself and $E\left( {d_{i} } \right)$ represents all directed edges from parent nodes to their respective children. The semantic value of disease $d_{i}$ is defined by

3 $${\text{DSV}}\left( {d_{i} } \right) = \mathop \sum \limits_{{d_{j} \in V\left( {d_{i} } \right)}} D_{{d_{i} }} \left( {d_{j} } \right)$$

where $D_{{d_{i} }} \left( {d_{j} } \right)$, as the semantic contribution value of disease $d_{j}$ to $d_{i}$, could be calculated as

4 $$D_{{d_{i} }} \left( {d_{j} } \right) = \left\{ {\begin{array}{*{20}l} {1,} \hfill & {if\; d_{j} = d_{i} } \hfill \\ {max\left\{ {\rho *D_{{d_{i} }} \left( {d_{j}^{^{\prime}} } \right)|d_{j}^{^{\prime}} \in children\; of\; d_{j} } \right\}, } \hfill & { otherwise} \hfill \\ \end{array} } \right.$$

where $\rho$ is a contribution factor. The semantic contribution value of disease $d_{j}$ to $d_{i}$ is inversely proportional to the distance between them in the DAG.

Based on the idea that two diseases will be more similar if their DAGs overlaps more nodes, we constructed semantic similarly matrix $SD$ for those diseases. Each element of $SD$ denotes the semantic similarly of disease pair $\left\langle {d_{k} ,d_{l} } \right\rangle$, which could be calculated as following:

5 $$SD\left( {d_{k} ,d_{l} } \right) = \frac{{\mathop \sum \nolimits_{{t \in V\left( {{\text{d}}_{k} } \right) \cap V\left( {{\text{d}}_{l} } \right)}} \left( {D_{{{\text{d}}_{k} }} \left( t \right) + D_{{{\text{d}}_{l} }} \left( t \right)} \right)}}{{{\text{DSV}}\left( {{\text{d}}_{k} } \right) + {\text{DSV}}\left( {{\text{d}}_{l} } \right)}}$$

### Gaussian kernel similarity for diseases and miRNAs

Gaussian kernel similarity comes from the topological distribution of the experimentally verified MDAs. Herein, we introduced binary vector ${ }BV\left( {d_{i} } \right)$ as the interaction profile for disease $d_{i}$, which is the $ith$ row of the adjacent matrix $X$. Hence, Gaussian kernel similarity of disease pair $< d_{i} ,d_{j} >$ could be expressed by

6 $$GD\left( {d_{i} ,d_{j} } \right) = {\text{exp}}\left( { - \delta_{d} BV\left( {d_{i} } \right) - BV\left( {d_{j} } \right)} \right)$$

where $\delta_{d}$ is a parameter for adjusting kernel bandwidth, which could be generated through averaging the interaction profiles of all diseases.

7 $$\delta_{d} = \frac{{\delta_{d}^{^{\prime}} }}{{\frac{1}{{N_{d} }}\mathop \sum \nolimits_{i = 1}^{{N_{d} }} BV\left( {d_{i} } \right)}}$$

In the same manner, Gaussian kernel similarity between miRNA pair $\left\langle {m_{i} ,m_{j} } \right\rangle$ could be defined as follows:

8 $$GM\left( {m_{i} ,m_{j} } \right) = {\text{exp}}\left( { - \delta_{m} BV\left( {m_{i} } \right) - BV\left( {m_{j} } \right)} \right)$$

9 $$\delta_{m} = \frac{{\delta_{m}^{^{\prime}} }}{{\frac{1}{{N_{m} }}\mathop \sum \nolimits_{i = 1}^{{N_{m} }} BV\left( {m_{i} } \right)}}$$

where $BV\left( {m_{i} } \right)$ is the interaction profile for miRNA $m_{i}$ and $\delta_{m}$ is used to control kernel bandwidth.

### Integrated similarities for miRNAs and diseases

Following the previous steps, the miRNA functional similarity, disease semantic similarity and Gaussian kernel similarity were generated separately. To cope with data sparsity and effectively utilize all kinds of similarities and correlations, we further constructed an integrated similarity matrix $ID$ for diseases and $IM$ for miRNAs respectively, which could be expressed as follows:

10 $$ID\left( {d_{i} ,d_{j} } \right) = \left\{ {\begin{array}{*{20}l} {SD\left( {d_{i} ,d_{j} } \right), } \hfill & { if\; SSD\left( {d_{i} ,d_{j} } \right) > 0} \hfill \\ {GD\left( {d_{i} ,d_{j} } \right), } \hfill & { otherwise } \hfill \\ \end{array} } \right.$$

11 $$IM\left( {m_{i} ,m_{j} } \right) = \left\{ {\begin{array}{*{20}l} {FM\left( {m_{i} ,m_{j} } \right), } \hfill & { if \;FSM\left( {m_{i} ,m_{j} } \right) > 0} \hfill \\ {GM\left( {m_{i} ,m_{j} } \right),} \hfill & { otherwise } \hfill \\ \end{array} } \right.$$

To ensure the equal importance, each similarity value should be normalized to the same interval before integration. The specific approach is to subtract the mean value and divide it by the standard deviation of the corresponding matrix.

### HCFMDA

**Fig. 1:**
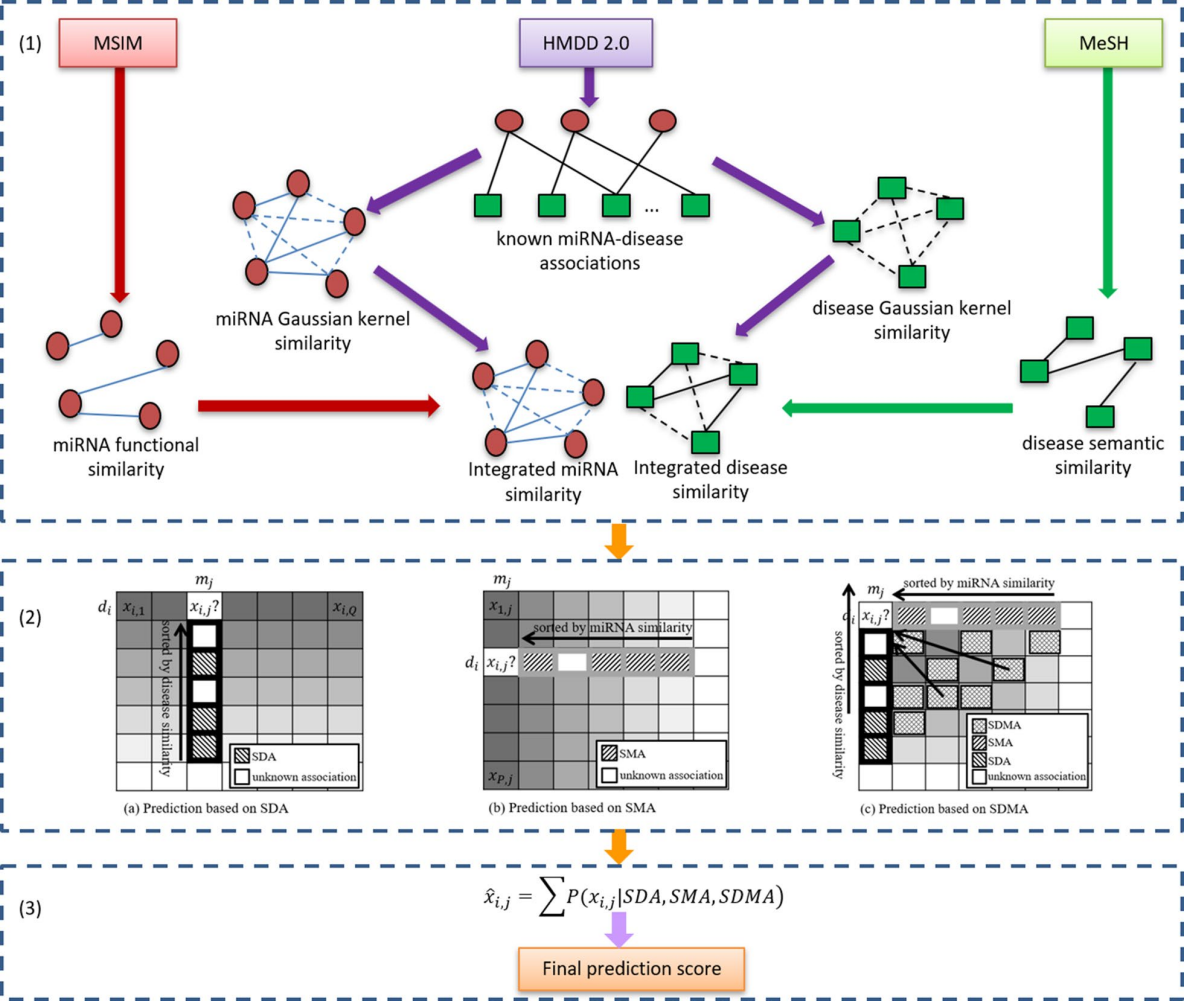
Flowchart of miRNA-disease association prediction based on HCFMDA. (1) Construct integrated similarities by incorporating the miRNA functional similarity, disease semantic similarity, Gaussian kernel similarity and experimentally verified MDA; (2) Calculate and fuse three different association sources: similar disease associations (SDA), similar miRNA associations (SMA), and similar disease-miRNA associations (SDMA); (3) Apply the proposed model to calculate the recommend score for test associations. Then, the predicted disease-related miRNAs could be further analyzed and experimentally validated

In this work, by integrating heterogeneous data including the miRNA functional similarity, disease semantic similarity, known MDA networks, Gaussian kernel similarity of miRNAs and diseases, we proposed a pipeline named improved hybrid collaborative filtering to predict miRNA-disease associations (HCFMDA). Figure 1 illustrates the flowchart of the entire process of HCFMDA. In order to fully exploit highly discriminative feature information embedded in the sparse MDAs, we incorporated and fused three different association sources: different diseases associated with the same miRNA, different miRNAs associated with the same disease, and ‘not-so-similar’ diseases or miRNAs, which could make the model more robust to data sparsity.

**Fig. 2:**
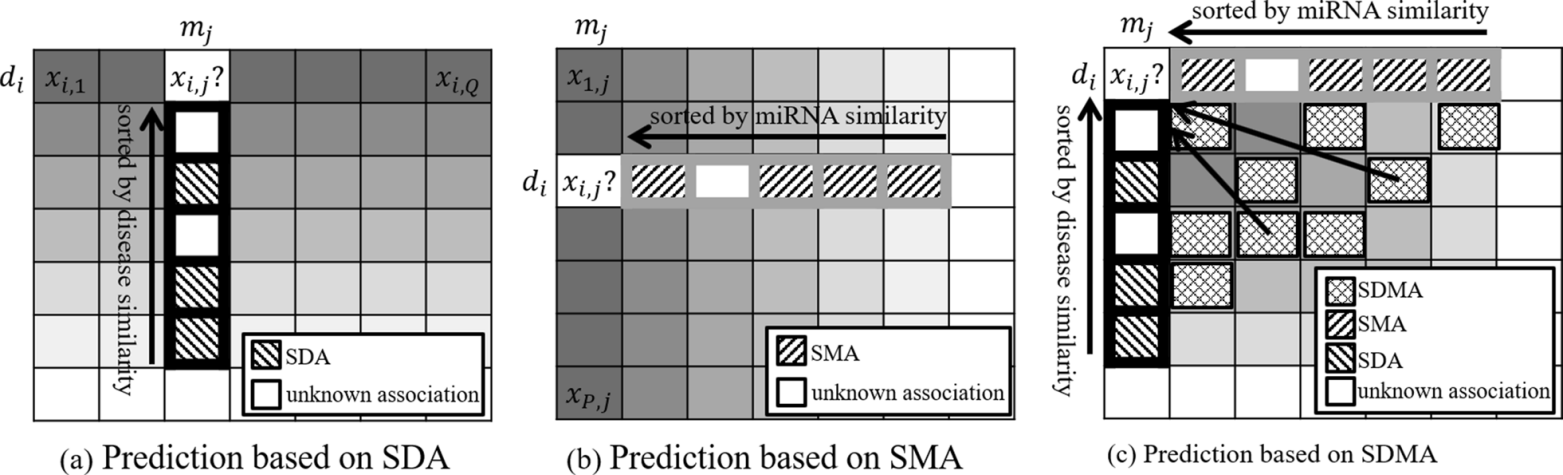
Three different prediction methods based on SDA, SMA and SDMA

Disease-based collaborative filtering predicted the score $\hat{x}_{i,j}$ of a test miRNA $m_{j}$ for a disease $d_{i}$ based on top-$N$ most similar diseases towards $d_{i}$. Consequently, the corresponding recommended score $\hat{x}_{i,j}$ could be represented by

12 $$\hat{x}_{i,j} = \frac{1}{N}\mathop \sum \limits_{{d_{u} \in T_{d} \left( {d_{i} } \right)}} s_{d} \left( {d_{i} ,d_{u} } \right)X_{u,j} , |T_{d} \left( {d_{i} } \right))| = N$$

where $T_{d} \left( {d_{i} } \right)$ and $s_{d} \left( {d_{i} ,d_{u} } \right)$ represent a set of top-$N$ most similar diseases towards disease $d_{i}$ and the integrated similarity value of disease pair $\left\langle {d_{i} , d_{u} } \right\rangle$, respectively. It could be seen from Fig. 2a that this method only exploits the known associations between the test miRNA and similar diseases of $d_{i}$, which accounts for only a small part in the matrix. We denoted this predictive source as the set of similar disease associations (SDA):

13 $${ }SDA_{i,j} = \left\{ {X_{u,j} |d_{u} \in T_{d} \left( {d_{i} } \right)} \right\}$$

Similarly, the predictive score $\hat{x}_{i,j }$ of test pair $\left\langle {d_{i} ,m_{j} } \right\rangle$ of miRNA-based collaborative filtering could also be calculated by averaging the associations of similar miRNAs related to the same disease $d_{i}$.

14 $$\hat{x}_{i,j} = \frac{1}{N}\mathop \sum \limits_{{m_{v} \in T_{m} \left( {m_{j} } \right)}} s_{m} \left( {m_{j} ,m_{v} } \right)X_{i,v} , |T_{m} \left( {m_{j} } \right))| = N$$

where $T_{m} \left( {m_{j} } \right)$ and $s_{m} \left( {m_{j} ,m_{v} } \right)$ denote a set of top-$N$ most similar miRNAs towards miRNA $m_{j}$ and the integrated similarity between miRNA $m_{j}$ and $m_{v}$, respectively. As illustrated in Fig. 2b, Eq. (15) only exploits the known similar miRNAs associated with the test disease for prediction. We refer to these predictive sources as the set of similar miRNA associations (*SMA*):

15 $${ }SMA_{i,j} = \left\{ {X_{i,v} |m_{v} \in T_{m} \left( {m_{j} } \right)} \right\}$$

In practice, solely relying on such SDA or SMA is undesirable, particularly when the association adjacent matrix $X$ is very sparse. The predictive accuracy could be improved by incorporating more associations from those ‘not-so-similar’ diseases or miRNAs. As illustrated in Fig. 2c, those associations from ‘not-so-similar’ diseases or miRNAs could provide additional information to improve the prediction. In this work, we refer to this predictive source as similar disease-miRNA associations (*SDMA*):

16 $$SDMA_{i,j} = \left\{ {X_{u,v} |d_{u} \in T_{d} \left( {d_{i} } \right), m_{v} \in T_{m} \left( {m_{j} } \right), u \ne i, v \ne j} \right\}$$

17 $$\hat{x}_{i,j} = \frac{1}{K}\mathop \sum \limits_{{X_{u,v} \in T_{d,m} \left( {X_{i,j} } \right)}} s_{d,m} \left( {X_{i,j} ,X_{u,v} } \right)X_{u,v} , |T_{d,m} \left( {X_{i,j} } \right))| = K$$

where $T_{d,m} \left( {X_{i,j} } \right)$ denotes a set of top-$K$ most similar miRNA-disease pairs. Here, we constructed $s_{d,m} \left( {X_{i,j} ,X_{u,v} } \right)$ as the similarity between entity $X_{i,j}$ and $X_{u,v}$.

18 $$s_{d,m} \left( {X_{i,j} ,X_{u,v} } \right) = \frac{1}{{\sqrt {\left( {1/s_{d} \left( {d_{i} ,d_{u} } \right)} \right)^{2} + \left( {1/s_{m} \left( {m_{j} ,m_{v} } \right)} \right)^{2} } }}$$

Each element of the matrix $X$ was employed as a separate predictor, whose confidence could be calculated according to its similarity towards the test association. We then predicted the expected value of the test association by averaging the individual predictions weighted by their confidence.

Finally, we calculated the expected value of the unknown test association $X_{i,j}$ by the following equation:

19 $$\begin{aligned} \hat{x}_{i,j} & = \sum P(X_{i,j} |SDA,SMA,SDMA) \\ & = \sum P\left( {X_{i,j} {|}SDA} \right)\alpha \left( {1 - \beta } \right) \\ & \quad + \sum P\left( {X_{i,j} {|}SMA} \right)\left( {1 - \alpha } \right)\left( {1 - \beta } \right) \\ & \quad + \sum P\left( {X_{i,j} {|}SDMA} \right)\beta \\ \end{aligned}$$

where $P(X_{i,j} |SDA,SMA,SDMA)$ denotes the estimating conditional probability depending on the predictors coming from the pool of $SDA$, $SMA$ and $SDMA$. Likewise, $P(X_{i,j} |SDA)$, $P(X_{i,j} |SMA)$, $P(X_{i,j} |SDMA)$ represent the pool of $SDA$, $SMA$ and $SDMA$ predictors, respectively. $\alpha$ and $\beta$ were used to control the selection (sampling) of data from those three different sources. If $\beta$ is equal 1, HCFMDA only uses SDMA recommend score to predict potential miRNAs for given diseases. In addition, to remove noise and accelerate the operation speed, singular value decomposition (SVD) technique was applied in HCFMDA.

### Performance evaluation

In practice, there are only 5430 experimentally verified MDAs (i.e., known associations) from HMDD V2.0 [28] and therefore most elements ($383{*}495 - 5430 = 184155$) in adjacent matrix $X$ are zeros, which indicates that $X$ is very sparse and so it is not feasible to adopt multi-fold cross-validation to test the performance of our method. As demonstrated in a series of studies [31–37], leave-one-out cross-validation (LOOCV) is more rigorous and objective than independent dataset test and K-fold cross-validation. Therefore, we implemented LOOCV to validate the performance of HCFMDA. In the LOOCV, for a designated disease ${ }d_{i}$, each known $d_{i}$-related association was left out in turn as a test sample and all other known associations (in total 5429) were used to train the model. Therefore, other miRNAs irrelevant to the disease $d_{i}$ along with the test miRNA were treated as candidate miRNAs. Then, we sorted all candidate miRNAs by the predictive scores derived from our model in descending order. If the rank of the test association exceeded a given threshold, we could view it as a successful identification. Receiver-operating characteristics (ROC) curve is a fundamental evaluation tool to illustrate diagnostic ability of a binary classifier. The ROC curve is generated by plotting true positive rate (TPR) against false positive rate (FPR) at different cut-off points. The corresponding formulas are as follows:

20 $$TPR = \frac{TP}{{TP + FN}}$$

21 $$FPR = \frac{FP}{{FP + TN}}$$

where $TP$, $FN$, $FP$ and $TN$ represents true positive, false negative, false positive and true negative, respectively. More specifically, $TP$ represents the number of known MDAs (positive samples) predicted correctly, and $FN$ is the number of positive samples that are falsely predicted to unknown MDAs (negative samples). Similarly, $FP$ denotes the number of negative samples incorrectly predicted to positive samples while $TN$ stands for the number of negative samples predicted correctly.

In addition, we calculated AUC to evaluate the predictive performance of HCFMDA. The AUC is equivalent to the probability that a classifier will rank a randomly chosen positive sample higher than a randomly chosen negative one. Specifically, AUC = 1 represents a perfect test, while AUC = 0.5 means a worthless test.

## Results

### Experimental results performed by HCFMDA

**Fig. 3:**
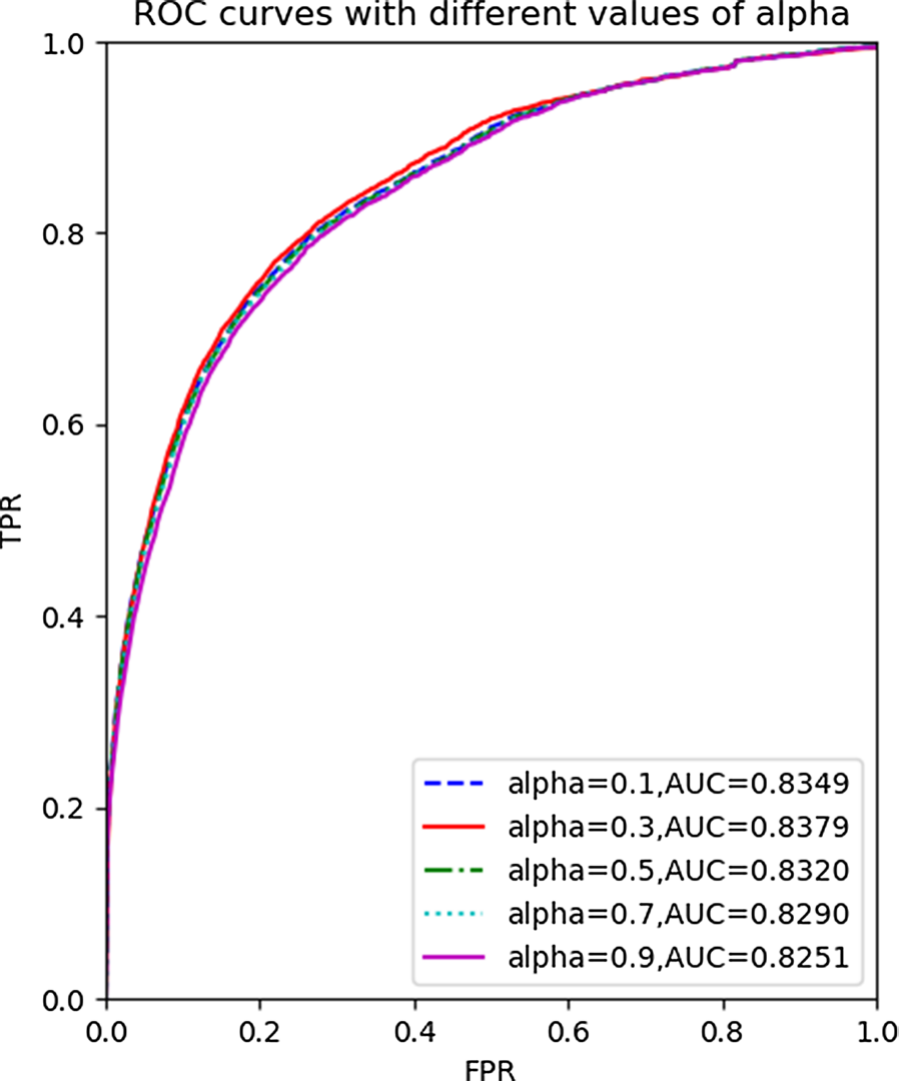
Optimal search for proportion factor $\upalpha$ performed by HCFMDA on HMDD v2.0 with $\upbeta = 0.1$

To verify the performance of HCFMDA to identify disease-related miRNAs, LOOCV was employed as testing strategy based on HMDD V2.0 dataset. For parameters $\upalpha$ and $\upbeta$, we adopted grid search strategy to search their optimal values ($\upalpha = 0.3,{ }\upbeta = 0.1$). Figure 3 illustrates the AUC values performed by HCFMDA on HMDD 2.0 by varying $\upalpha$ from 0.1 to 1.0 with the step of 0.2, where $\upbeta$ is set to 0.1. It indicates that the fusion of multi-similarity measurement could enhance the prediction performance of HCFMDA model.

**Fig. 4:**
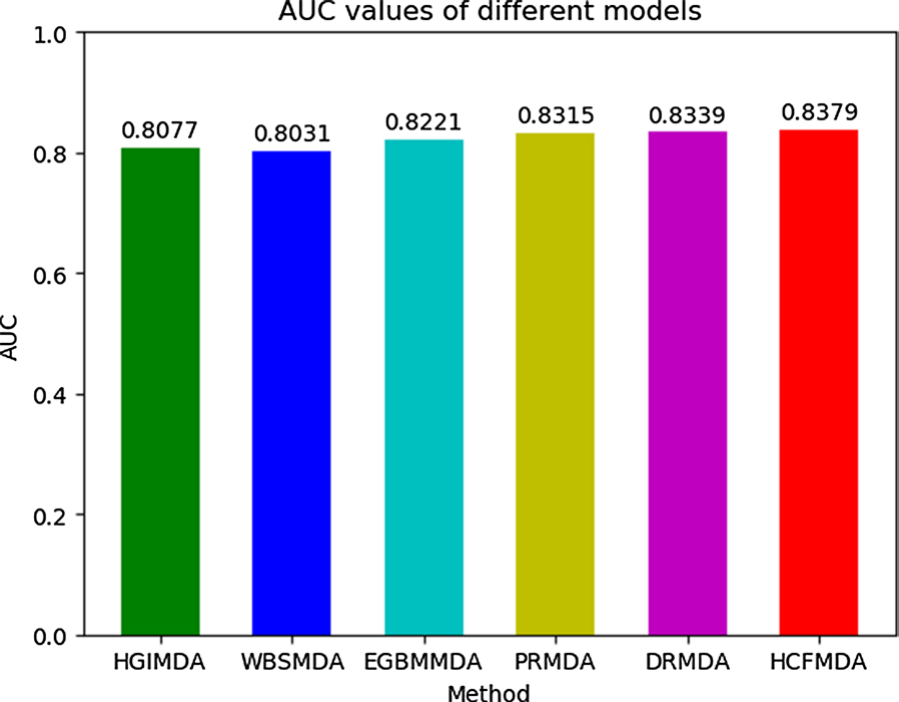
AUC values of HCFMDA and five other disease-miRNA prediction models

Then, we compared HCFMDA with other five state-of-the-art computational approaches: HGIMDA [20], WBSMDA [38], EGBMMDA [39], PRMDA [21] and DRMDA [40]. Figure 4 illustrates the AUC values comparison of those prediction models in the same framework of LOOCV. Our model achieved AUC of 0.8379, while the AUC values from HGIMDA, WBSMDA, EGBMMDA, PRMDA and DRMDA were 0.8077, 0.8031, 0.8221, 0.8315 and 0.8339, respectively. Because HCFMDA could capture high discriminative information embedded in the correlations between miRNAs and diseases, it achieved the superior predictive performance compared with the other five methods. Besides, the ROC curve of HCFMDA is smoother than those of other methods, which reflects HCFMDA is more robust and accurate. In addition, the time complexity of our model is obvious lower than the other methods. We attribute it to the introduction of SVD matrix decomposition that could reduce the impact of noise and improve the predictive speed. In conclusion, HCFMDA demonstrates reliable performance for predicting MDAs.

### Case validation 1: for diseases with known associated miRNAs

Apart from the validation of LOOCV, we also carried out case studies for several important human diseases to further verify the performance of HCFMDA. Predictive results of HCFMDA were confirmed by matching experimentally verified MDAs (i.e., known associations) from two other independent databases: miR2Disease [41] and dbDEMC [42]. Herein, we adopted two types of case validations. At first, we applied HCFMDA over three different diseases, i.e. Colon Neoplasms (CN), Esophageal Neoplasms (EN), and Prostate Neoplasms (PN). For a given disease, each time a known association in HMDD v2.0 was left out as a test sample and all unknown associations were taken as the candidate miRNAs, while all other known associations were used for training samples. In the second type of case validation for Breast Neoplasms (BN), we removed all known associations related to BN in HMDD v2.0 and then employed HCFMDA to predict potential BN-related miRNAs. The key point of this type of case validation is to ensure that the prioritization of putative miRNAs related to BN only makes use of the correlations of other phenotypic diseases similar to BN. Therefore, it could be used to demonstrate whether our model is applicable to those isolated diseases without any known related miRNAs.

As the most common type of gastrointestinal cancer, CN is the second-leading cause of cancer-related deaths in the USA. It was estimated that about 140,250 people was diagnosed with CN, and 50,630 died from the disease in 2018 [43]. Since patients in early stage of CN have only mild symptoms and are hard to be detected, there is an urgent demand of novel diagnostic biomarkers for its early detection. Fortunately, a significant number of CN-related miRNAs have been successfully identified in the past decades. For instance, Ma et al*.* [44] reported that the up-regulated miRNAs in CN including miR-182, miR-17, miR-106a, miR-93, miR-200c, miR-92a, let-7a and miR-20a (FDR value < 5%), while the downregulated miRNAs were miR-l195, miR-143 and miR-145 (FDR value < 5%). Moreover, Marta et al*.* [45] identified and validated a signature of 6 miRNAs (miRNA19a, miRNA19b, miRNA15b, miRNA29a, miRNA335, and miRNA18a) as biomarkers that could differentiate significantly CN patients from those healthy people. In the case validation for CN, we adopted HCFMDA to prioritize the top 50 miRNAs from candidate miRNAs (Table 1). We found that 9 out of the top 10 candidate miRNAs could be supported to be associated with CN by the experimental evidence. Besides, 88% of top 50 prioritized miRNAs were validated to be involved with CN. For example, many experiments [46] reported that the expression level of has-mir-20a (ranked No.1 in Table 1) was significantly higher in CN tissue than those in the normal adjacent mucosa and suggests that it can be taken as a novel prognostic marker and therapeutic target for CN. Also, miR-18a (ranked No.2 in Table 1) was also confirmed by experiments and could play an important role in CN pathogenesis [47]. Actually, some of the rest unconfirmed miRNAs in Table 1 were also confirmed by recent studies. For example, hsa-mir-92 directly targeted the anti-apoptotic molecule BCL-2-interacting mediator of cell death (BIM) in colon cancer tissues and was recently proposed as a key oncogenic component of miR-17–92 cluster through targeting and down-regulating the proapoptotic protein Bim in CN [48]. In addition, Antonio et al. [49] reported that has-miR-101 expression was differentially impaired in CN specimens and it might function as a tumor suppressor in CN and that its pharmacological restoration might hamper the aggressive behavior of CRC in vivo.

**Table 1:** Predictive results of the top 50 prioritized miRNAs related to CN based on experimentally verified associations in HMDD v2.0 database

miRNA	Evidence	miRNA	Evidence
hsa-mir-20a	dbDEMC, miR2Disease	hsa-mir-92a	Unconfirmed
hsa-mir-18a	dbDEMC, miR2Disease	hsa-mir-141	dbDEMC, miR2Disease
hsa-mir-19b	dbDEMC, miR2Disease	hsa-mir-214	dbDEMC
hsa-mir-191	dbDEMC, miR2Disease	hsa-mir-30c	dbDEMC, miR2Disease
hsa-mir-143	dbDEMC, miR2Disease	hsa-mir-93	dbDEMC, miR2Disease
hsa-mir-132	miR2Disease	hsa-mir-34c	miR2Disease
hsa-mir-29b	dbDEMC, miR2Disease	hsa-mir-21	dbDEMC, miR2Disease
hsa-mir-19a	dbDEMC, miR2Disease	hsa-mir-25	dbDEMC, miR2Disease
hsa-mir-34a	dbDEMC, miR2Disease	hsa-mir-194	dbDEMC, miR2Disease
hsa-mir-101	Unconfirmed	hsa-mir-32	dbDEMC, miR2Disease
hsa-let-7e	dbDEMC	hsa-mir-92b	Unconfirmed
hsa-let-7d	dbDEMC	hsa-mir-205	dbDEMC
hsa-let-7a	dbDEMC, miR2Disease	hsa-let-7 g	dbDEMC, miR2Disease
hsa-mir-200b	dbDEMC	hsa-mir-222	dbDEMC
hsa-mir-127	dbDEMC, miR2Disease	hsa-mir-203	dbDEMC, miR2Disease
hsa-mir-125b	dbDEMC	hsa-mir-146a	dbDEMC
hsa-mir-199a	Unconfirmed	hsa-mir-34b	dbDEMC, miR2Disease
hsa-mir-223	dbDEMC, miR2Disease	hsa-mir-16	dbDEMC
hsa-let-7b	dbDEMC, miR2Disease	hsa-mir-429	dbDEMC
hsa-mir-125a	dbDEMC, miR2Disease	hsa-mir-221	dbDEMC, miR2Disease
hsa-mir-155	dbDEMC, miR2Disease	hsa-mir-200a	Unconfirmed
hsa-let-7c	dbDEMC	hsa-mir-146b	Unconfirmed
hsa-mir-106b	dbDEMC, miR2Disease	hsa-mir-29a	dbDEMC, miR2Disease
hsa-let-7f	dbDEMC, miR2Disease	hsa-mir-95	dbDEMC, miR2Disease
hsa-let-7i	dbDEMC	hsa-mir-373	dbDEMC

The column 1 and 3 list the top 1–25 and top 26–50 CN-related miRNAs, respectively

Esophageal neoplasms (EN), or esophageal cancer, occupies the sixth position among malignant tumors worldwide with regard to mortality and ranks fourth in China [50]. Due to lack of effective clinical diagnosis approaches for EN, it is often diagnosed at a more advanced stage and its overall 5-year survival rate is only about 25% [51]. Therefore, investigating the mechanism of EN is seriously essential to improve its diagnosis, treatment and prognosis. Numerous recent studies have indicated that aberrant expression of miRNAs is involved in EN. For instance, Hu et al*.* [50] identified that miR‑375 was downregulated in tumor tissue and cell line EC109 of EN samples when compared with normal tissues and cells. Experiments confirmed that as a tumor suppressor in EN cells, miR-375 inhibited cell proliferation and invasion by repressing the expression of its direct target MTDH, an oncogene associated with tumorigenesis in EN. Herein, we took EN as a case validation and prioritized the candidate miRNAs of the disease. As illustrated in Table 2, all the top 10 predicted miRNAs associated with EN were successfully verified by experimental evidence collected from the two independent databases. Meanwhile, 46 out of the top 50 predicted miRNAs were also validated to be related to EN. For example, recent studies indicated that miR-195 was down-regulated in EN tissues compared with normal esophageal tissues ($P$ = 0.05) and experimental results indicated that Cdc42 protein was reduced after miR-195 mimics transfected ($P = 0.01$) [52]. In addition, Zhang et al*.* [53] first presented that tanshinone IIA inhibited human EN cell growth through miR-122-mediated Pyruvate kinase M2 (PKM2) down-regulation pathway.

**Table 2:** Predictive results of the top 50 predicted miRNAs related to EN based on known associations in HMDD v2.0 database

miRNA	Evidence	miRNA	Evidence
hsa-mir-17	dbDEMC	hsa-mir-24	dbDEMC
hsa-mir-18a	dbDEMC	hsa-mir-10b	dbDEMC
hsa-mir-19b	dbDEMC	hsa-mir-30c	dbDEMC
hsa-mir-125b	dbDEMC	hsa-mir-30a	dbDEMC
hsa-mir-221	dbDEMC	hsa-mir-181a	dbDEMC
hsa-mir-16	dbDEMC	hsa-mir-15b	dbDEMC
hsa-mir-29a	dbDEMC	hsa-mir-93	dbDEMC
hsa-mir-200b	dbDEMC	hsa-mir-106a	dbDEMC
hsa-mir-106b	dbDEMC	hsa-mir-18b	dbDEMC
hsa-let-7d	dbDEMC	hsa-mir-132	dbDEMC
hsa-mir-1	dbDEMC	hsa-mir-23b	dbDEMC
hsa-let-7i	dbDEMC	hsa-mir-122	unconfirmed
hsa-let-7f	unconfirmed	hsa-mir-194	dbDEMC,miR2Disease
hsa-let-7e	dbDEMC	hsa-mir-7	dbDEMC
hsa-mir-222	dbDEMC	hsa-mir-218	unconfirmed
hsa-mir-29b	dbDEMC	hsa-mir-127	dbDEMC
hsa-mir-429	dbDEMC	hsa-mir-302c	dbDEMC
hsa-mir-181b	dbDEMC	hsa-mir-199b	dbDEMC
hsa-mir-142	dbDEMC	hsa-mir-135a	dbDEMC
hsa-mir-125a	dbDEMC	hsa-mir-193b	dbDEMC
hsa-mir-182	dbDEMC	hsa-mir-20b	dbDEMC
hsa-let-7g	dbDEMC	hsa-mir-302b	dbDEMC
hsa-mir-195	dbDEMC	hsa-mir-107	dbDEMC,miR2Disease
hsa-mir-146b	dbDEMC	hsa-mir-204	unconfirmed
hsa-mir-9	dbDEMC	hsa-mir-23a	dbDEMC

The column 1 and 3 list the top 1–25 and top 26–50 EN-related miRNAs, respectively

Prostate neoplasms (PN) is the most common malignancy and the third leading cancer-related cause of death among men in the western world. Although the 5-year survival rate of PN is higher in early-stage after treatment with surgical resection or androgen deprivation therapy, one-third of treated PN patients will experience disease recurrence and progress into castration-resistant PN, a more aggressive disease [54]. Therefore, an impressing need exists to identify novel miRNAs as tools or biomarkers for the prediction of aggressive PN. In the case validation for PN by HCFMDA, 8 of top 10 miRNAs and 44 out of top 50 candidate PN-associated miRNAs were validated by the two independent databases (see Table 3). Moreover, 4 of the rest 6 unsupported miRNAs were verified by recent studies. For example, Williams et al*. *[55] identified miR-200b as a downstream target of androgen receptor and linked its expression to decreased tumorigenicity and metastatic capacity of the prostate cancer cells. In addition, as an "antimetastatic" miRNA in PN, miR-203 expression is specifically attenuated in bone metastatic prostate cancer suggesting a fundamental antimetastatic role for this miRNA [56].

**Table 3:** Predictive results of the top 50 prioritized miRNAs related to PN based on known associations in HMDD v2.0 database

miRNA	Evidence	miRNA	Evidence
hsa-mir-21	dbDEMC,miR2Disease	hsa-mir-200a	dbDEMC
hsa-mir-155	dbDEMC	hsa-mir-23a	dbDEMC,miR2Disease
hsa-let-7a	dbDEMC,miR2Disease	hsa-mir-106b	dbDEMC
hsa-mir-146a	miR2Disease	hsa-mir-19b	dbDEMC,miR2Disease
hsa-mir-17	miR2Disease	hsa-mir-24	dbDEMC,miR2Disease
hsa-mir-20a	miR2Disease	hsa-let-7b	dbDEMC,miR2Disease
hsa-mir-143	dbDEMC,miR2Disease	hsa-mir-223	dbDEMC,miR2Disease
hsa-mir-18a	24752237	hsa-mir-34a	dbDEMC,miR2Disease
hsa-let-7c	dbDEMC,miR2Disease	hsa-mir-15a	dbDEMC,miR2Disease
hsa-mir-92a	29568403	hsa-let-7i	dbDEMC
hsa-mir-181b	dbDEMC,miR2Disease	hsa-mir-200b	24391862
hsa-let-7f	dbDEMC,miR2Disease	hsa-mir-25	dbDEMC,miR2Disease
hsa-mir-19a	dbDEMC	hsa-mir-142	unconfirmed
hsa-mir-1	dbDEMC	hsa-mir-141	miR2Disease
hsa-mir-9	dbDEMC	hsa-mir-222	dbDEMC,miR2Disease
hsa-mir-126	dbDEMC,miR2Disease	hsa-let-7g	dbDEMC,miR2Disease
hsa-let-7e	dbDEMC	hsa-mir-29c	dbDEMC
hsa-mir-221	dbDEMC,miR2Disease	hsa-mir-125a	dbDEMC,miR2Disease
hsa-let-7d	dbDEMC,miR2Disease	hsa-mir-203	21159887
hsa-mir-16	dbDEMC,miR2Disease	hsa-mir-106a	dbDEMC,miR2Disease
hsa-mir-150	dbDEMC	hsa-mir-133a	dbDEMC
hsa-mir-29a	dbDEMC,miR2Disease	hsa-mir-34b	dbDEMC
hsa-mir-93	26124181	hsa-mir-34c	dbDEMC
hsa-mir-210	miR2Disease	hsa-mir-27a	dbDEMC,miR2Disease
hsa-mir-200c	dbDEMC	hsa-mir-15b	dbDEMC

The column 1 and 3 list the top 1–25 and top 26–50 PN-related miRNAs, respectively. The evidences for the associations are either database studies or PMIDs of other experimental literatures

Besides, we further made the comparison between HCFMDA and other five aforementioned MDA prediction models in terms of the case studies of diseases CN, EN and PN. It could be seen from Table 4 that HCFMDA ranks second best among all predictive models for those three diseases. The predictive hit rate of HCFMDA is only lower than that of PRMDA in the case study of CN and EN, and that of EGBMMDA in the case study of PN, which fully demonstrates that HCFMDA could be used as a reliable tool for predicting disease-related miRNAs.

**Table 4:** Comparison results of the first case study by HCFMDA and other five state-of-the art predictive models

Disease	HGIMDA	WBSMDA	EGBMMDA	PRMDA	DRMDA	HCFMDA
Colon neoplasms	45	45	43	46	44	44
Esophageal neoplasms	44	NULL	NULL	47	NULL	46
Prostate neoplasms	44	40	45	43	43	44

NULL denotes the corresponding model did not performed the case study for the designated disease

### Case validation 2: for diseases without known associated miRNAs

To further validate the predictive performance of HCFMDA for those diseases without any known related miRNA, we also implemented another type of case validation for Breast Neoplasms (BN) by removing all the known BN-related associations in HMDD v2.0. That is to say, we only utilized the known associations of other diseases except BN and adopted the indirect way to predict BN-related miRNAs. We then ranked all the 495 candidate miRNAs by their predictive scores and verified the top 50 ones according to the databases of dbDEMC, miR2Disease and HMDD v2.0. The predictive results (Table 5) indicated that all top 10 and 46 out of the top 50 prioritized miRNAs were confirmed by those databases. The achieved results indicate that HCFMDA could also be applied to predict novel miRNAs for those isolated diseases.

**Table 5:** Predictive results of the top 50 prioritized miRNAs related to BN through removing all known BN-related miRNAs in HMDD V2.0 database

miRNA	Evidence	miRNA	Evidence
hsa-mir-367	dbDEMC,HMDD	hsa-mir-608	dbDEMC,HMDD
hsa-mir-302c	dbDEMC,HMDD	hsa-mir-638	dbDEMC,HMDD
hsa-mir-302a	dbDEMC,HMDD	hsa-mir-518b	unconfirmed
hsa-mir-302b	dbDEMC,HMDD	hsa-mir-602	dbDEMC
hsa-mir-488	HMDD	hsa-mir-612	dbDEMC
hsa-mir-215	dbDEMC,HMDD	hsa-mir-615	dbDEMC
hsa-mir-302d	dbDEMC,HMDD	hsa-mir-637	dbDEMC
hsa-mir-218	dbDEMC,HMDD	hsa-mir-657	dbDEMC
hsa-mir-383	dbDEMC,HMDD	hsa-mir-185	dbDEMC
hsa-let-7d	dbDEMC,miR2Disease,HMDD	hsa-mir-518c	dbDEMC
hsa-let-7f	dbDEMC,miR2Disease,HMDD	hsa-mir-622	dbDEMC
hsa-let-7c	dbDEMC,HMDD	hsa-mir-583	dbDEMC
hsa-mir-19a	dbDEMC,HMDD	hsa-mir-557	dbDEMC
hsa-mir-153	dbDEMC,HMDD	hsa-mir-600	dbDEMC
hsa-let-7b	dbDEMC,HMDD	hsa-mir-601	dbDEMC
hsa-let-7i	dbDEMC,miR2Disease,HMDD	hsa-mir-611	unconfirmed
hsa-mir-296	dbDEMC,HMDD	hsa-mir-654	dbDEMC
hsa-let-7e	dbDEMC,HMDD	hsa-mir-662	dbDEMC
hsa-let-7a	dbDEMC,miR2Disease,HMDD	hsa-mir-769	unconfirmed
hsa-mir-429	dbDEMC,miR2Disease,HMDD	hsa-mir-18a	dbDEMC,miR2Disease,HMDD
hsa-mir-338	dbDEMC,HMDD	hsa-mir-486	dbDEMC,HMDD
hsa-let-7g	dbDEMC,HMDD	hsa-mir-629	dbDEMC,HMDD
hsa-mir-20a	miR2Disease,HMDD	hsa-mir-596	unconfirmed
hsa-mir-19b	dbDEMC,HMDD	hsa-mir-17	miR2Disease,HMDD
hsa-mir-324	HMDD	hsa-mir-339	dbDEMC,HMDD

The column 1 and 3 list the top 1–25 and top 26–50 BN-related miRNAs, respectively

## Discussion

Identification of novel disease-related miRNAs is beneficial for understanding disease pathogenesis at the molecular level, and developing effective disease diagnostic biomarkers and therapeutic tools. In this work, we proposed an efficient computational framework, HCFMDA, to improve the predictive performance of MDAs by integrating heterogeneous information: miRNA functional similarity, disease semantic similarity, known MDA networks, Gaussian kernel similarity of miRNAs and diseases. HCFMDA employs not only traditional disease-based and miRNA-based associations, but also associations from other ‘not-so-similar’ diseases and miRNAs to smooth the predictions. We then implemented LOOCV and two types of case validations over four important human cancers. The achieved results demonstrate that HCFMDA is indeed robust against data sparsity, which is better than other five state-of-the-art models, i.e., HGIMDA, WBSMDA, EGBMMDA, PRMDA, and DRMDA.

## Conclusions

The excellent performance of HCFMDA mainly attributes to the following aspects. First, many kinds of heterogeneous data including miRNA functional similarity, disease semantic similarity, and known MDAs were integrated into our model, which contains highly discriminative information. Second, by fusing three kinds of similar associations including disease-based associations, miRNA-based associations, and other ‘not-so-similar’ diseases and miRNAs associations our model could fully mine and capture the intrinsic associations between miRNAs and diseases even if the MDA matrix is very sparse. Although some favorable results have been made, there still exists several limitations in HCFMDA. First, there are only 5430 known MDAs among 383 diseases and 495 miRNAs, and therefore the corresponding MDA matrix is very sparse and needs to be further enriched. Second, although we have integrated some heterogeneous data into our model, there is still room for improving the performance of HCFMDA by integrating more effective data sources which could provide more useful information for predicting MDAs. Moreover, we will improve the efficiency of our model by introducing graph-based recommendation filtering algorithms in the future.

## Data Availability

The datasets used and/or analyzed during the current study are available at https://github.com/ivantsinghua/HCFMDA.

## Abbreviations

MDA: MiRNA-disease association
HCF: Hybrid collaborative filtering
LOOCV: Leave-one-out cross-validation
BV: Binary vector
DAG: Directed acyclic graph
SDA: Similar disease associations
SMA: Similar miRNA associations
SDMA: Similar disease-miRNA associations
SVD: Singular value decomposition
ROC: Receiver operating characteristic
AUC: Area under curve
FPR: False positive rate
TPR: True positive rate
TP: True positive
FN: False negative
FP: False positive
TN: True negative
